# Biological Effect of a Hybrid Anticancer Agent Based on Kinase and Histone Deacetylase Inhibitors on Triple-Negative (MDA-MB231) Breast Cancer Cells

**DOI:** 10.3390/ijms17081235

**Published:** 2016-07-30

**Authors:** Mariangela Librizzi, John Spencer, Claudio Luparello

**Affiliations:** 1 Dipartimento di Scienze e Tecnologie Biologiche, Chimiche, Farmaceutiche (STEBICEF), Edificio 16, Università di Palermo, Viale delle Scienze, Palermo 90128, Italy; merylib@alice.it; 2 Department of Chemistry, School of Life Sciences, University of Sussex, Falmer, Brighton BN1 9QJ, UK; j.spencer@sussex.ac.uk

**Keywords:** breast cancer, MDA-MB231 cells, histone deacetylase inhibitor, vascular endothelial growth factor receptor-2 inhibitor, cytotoxicity, cell cycle, apoptosis, autophagy, mitochondrial metabolism

## Abstract

We examined the effects of the histone deacetylase inhibitor (HDACi) suberoylanilide hydroxamic acid (SAHA) combined with the vascular endothelial growth factor receptor-1/2 inhibitor (3*Z*)-5-hydroxy-3-(1*H*-pyrrol-2-ylmethylidene)-2,3-dihydro-1*H*-indol-2-one on MDA-MB-231 breast cancer cells (triple-negative) in the form of both a *cocktail* of the separate compounds and a chemically synthesized hybrid (*N*-hydroxy-*N'*-[(3*Z*)-2-oxo-3-(1*H*-pyrrol-2-ylmethylidene)-2,3-dihydro-1*H*-indol-5-yl]octanediamide). Comparative flow cytometric and Western blot analyses were performed on *cocktail*- and hybrid-treated cells to evaluate cell cycle distribution, autophagy/apoptosis modulation, and mitochondrial metabolic state in order to understand the cellular basis of the cytotoxic effect. Cell cycle analysis showed a perturbation of the rate of progression through the cycle, with aspects of redistribution of cells over different cycle phases for the two treatments. In addition, the results suggest that the two distinct classes of compounds under investigation could induce cell death by different preferential pathways, i.e., autophagy inhibition (the *cocktail*) or apoptosis promotion (the hybrid), thus confirming the enhanced potential of the hybrid approach vs. the combination approach in finely tuning the biological activities of target cells and also showing the hybrid compound as an additional promising drug-like molecule for the prevention or therapy of “aggressive” breast carcinoma.

## 1. Introduction

**Figure 1:**
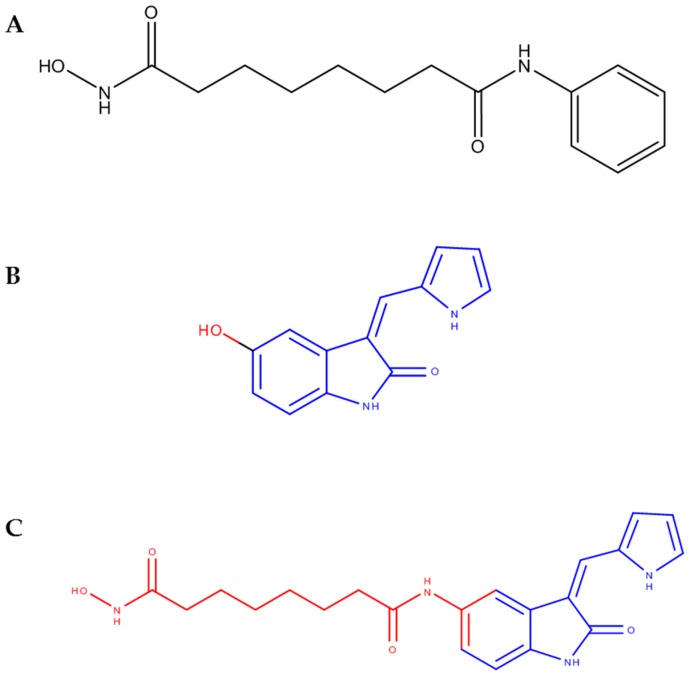
Compounds used in the present study. (**A**) SAHA (**1**), (**B**) (3*Z*)-5-hydroxy-3-(1*H*-pyrrol-2-ylmethylidene)-2,3-dihydro-1*H*-indol-2-one (**2**); (**C**) *N*-hydroxy-*N'*-[(3*Z*)-2-oxo-3-(1*H*-pyrrol-2-ylmethylidene)-2,3-dihydro-1*H*-indol-5-yl]octanediamide (**3**). SAHA, suberoylanilide hydroxamic acid.

It is generally acknowledged that several signaling pathways are involved in the aggressiveness and metastatic potential of malignant tumors. Therefore, the multifactorial nature of cancer illustrates the need for multifunctional therapeutic tools, such as employing the use of more than one compound to modulate different pathways. Combination therapy has been implemented by combining compounds in a “*cocktail*” of two or more unmodified molecules in a single solution, whereas the production of hybrid compounds is another emerging strategy which has gained popularity in the last decade [1,2]. In a previous publication, we [3] reported the synthesis of a hybrid drug (*N*-hydroxy-*N'*-[(3*Z*)-2-oxo-3-(1*H*-pyrrol-2-ylmethylidene)-2,3-dihydro-1*H*-indol-5-yl]octanediamide) based on the merging of fragments of the histone deacetylase inhibitor (HDACi) suberoylanilide hydroxamic acid (SAHA) and the vascular endothelial growth factor-1 and -2 receptor inhibitor (VEGFR1/2i) (3*Z*)-5-hydroxy-3-(1*H*-pyrrol-2-ylmethylidene)-2,3-dihydro-1*H*-indol-2-one (Figure 1), which was effective in reducing the viability of MDA-MB-231 cells, in an in vitro model system for triple-negative breast cancer (TNBC).

It is known that, due to a lack of expression of estrogen, progesterone and epidermal growth factor receptor by TNBC cells, this neoplastic cytotype is extremely “aggressive”; moreover, it is endowed with a higher malignant potential than other breast tumor subtypes [4]. Further, the limitation of treatment options has prompted the development of novel drugs or analogues of pre-existing drugs in the attempt to counteract TNBC cell growth. These compounds, on the other hand, necessitate a thorough biological evaluation. Experimental evidence has shown that the two parental molecules tested by us [3] are active in restraining MDA-MB-231 cell survival and growth. Compound **1** was found to affect the cell cycle and promote apoptosis [5], induce polyploidy-dependent senescence [6], and inhibit epidermal growth factor receptor (EGFR) expression, thereby disrupting the associated downstream signaling [7]. The combination of **1** with other drugs in hybrid anti-cancer molecules has proven to be effective, e.g., as reported by Mendoza-Sanchez et al. [8] who developed a bifunctional anti-proliferative **1**/estrogen receptor modulator ICI-164,384 compound active on both TNBC (MDA-MB-231) and estrogen receptor–positive (MCF-7) cells. VEGF is a renowned angiogenic growth factor that is recognized by a family of receptor tyrosine kinases, the VEGFRs, thereby promoting endothelial cell proliferation and migration [9]. In addition, it is known that VEGFRs are able to control the biological behavior of other non-endothelial cytotypes, including TNBC cells such as MDA-MB-231, thereby showing the potential to directly monitor tumor cell survival and the development of breast cancer [10]. In particular, the inhibition of VEGFR2 phosphorylation activity, which is directed to switch on the intracellular signalization of phosphatidylInositol 3-kinase (PI3K), AKT and signal transducer and activator of transcription 3 (STAT3), by the natural products xanthatin (a sesquiterpene lactone) and rhamnazin (an *O*-methylated flavonol), has been shown to significantly inhibit MDA-MB-231 cell growth both in culture and in nude mice [11,12].

In our previous work [3], both the **1/2** *cocktail* and compound **3** were shown to be cytotoxic on TNBC cells with an approximate 1:3 ratio of their half maximal inhibitory concentration (IC_50_) at 72 h. On the other hand, although comparable in their final effects on MDA-MB-231 cell survival, the different dose-response curves for the two treatments suggested a likely distinction, at least partially, of their biochemical and molecular mechanisms of action. Since characterization of the biological properties of new drugs is a key point for the evaluation of drugs’ potency within the composite intracellular microenvironment, the present work was aimed at acquiring the initial experimental data on the functional properties of the compounds under study when administered to MDA-MB-231 cell cultures through a panel of flow cytometric and immunoblot assays.

## 2. Results

In order to obtain comparative information on biological parameters of the cytotoxic action of the **1**/**2** *cocktail* and **3** on triple-negative MDA-MB-231 cells, cell cycle state, apoptosis induction markers (phosphatydilserine externalization and caspase-8 activation), mitochondrial metabolism and cell redox state markers (mitochondrial transmembrane potential (MMP) and reactive oxygen species (ROS) production), and autophagy markers (acidic vesicular organelle (AVO) and beclin-1 accumulation) were investigated.

**Figure 2:**
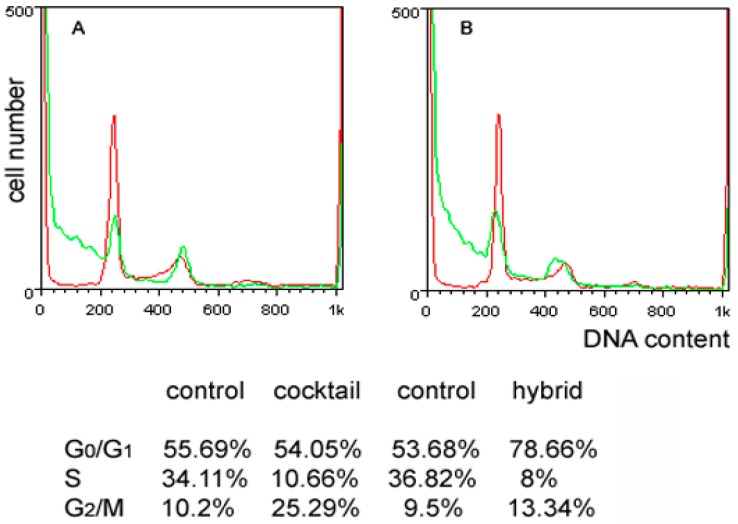
Effect of the **1**/**2** *cocktail* and **3** on the MDA-MB-231 cell cycle. DNA profiles of MDA-MB-231 cells following 72 h of culture under control conditions (**red** line in **A**,**B**) and in the presence of either 10 μM **1**/**2** *cocktail* (**green** line in **A**) or 29 μM **3** (**green** line in **B**). Cell distribution in the different cycle phases is reported in the Table (annex).

First, MDA-MB-231 cells were examined for distribution of cell cycle phases, and the results obtained are shown in Figure 2. Exposure to **3** induced a more prominent increase of the G_0_/G_1_ phase fraction than that recorded for the **1**/**2** *cocktail* (control vs. **1**/**2** *cocktail* = 55.69% vs. 64.05%; control vs. **3** = 53.68% vs. 78.66%), indicative of a more pronouncedly restrained progression via the S phase due to the conceivable activation of the corresponding checkpoint. In both experimental conditions, a similar marked decrease of the S phase fraction (control vs. **1**/**2** *cocktail* = 34.11% vs. 10.66%; control vs. **3** = 36.82% vs. 8%) was observed, a result that appears noteworthy since in breast cancer this fraction is regarded as prognostic. Moreover, an accumulation of cells in the G_2_/M phase (control vs. **1**/**2** *cocktail* = 10.2% vs. 25.29%; control vs. **3** = 9.5% vs. 13.34%), more conspicuous for the *cocktail* treatment and indicative of the inhibition of cell division, was also recorded.

**Figure 3:**
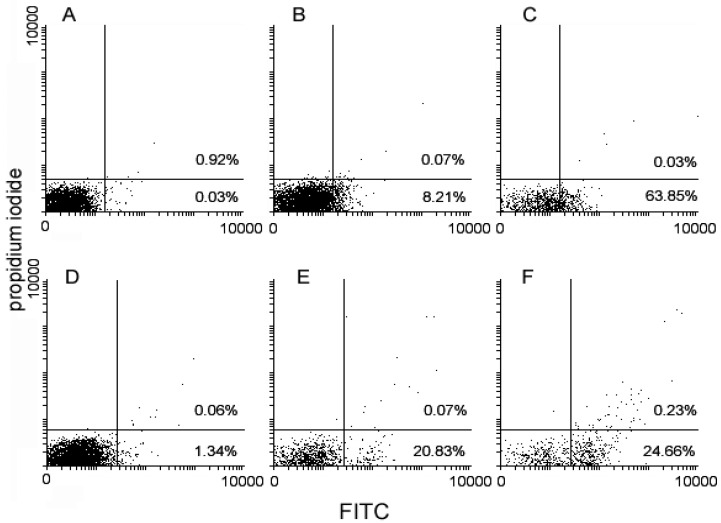
Flow cytometric analysis of control (**A**,**D**), **1**/**2** *cocktail*–treated (**B**,**E**) and **3**-treated MDA-MB-231 cells (**C**,**F**) stained with annexin V-FITC and propidium iodide for phosphatydilserine externalization (**A**–**C**), and with FAM-LETD-FMK caspase-8 reagent for caspase-8 activation (**D**–**F**). The percentage in the quadrants of plots **A**, **B** and **C** relates to late apoptotic/necrotic annexin V^+^/propidium iodide^+^ cells (top right quadrant) and early apoptotic annexin V^+^/propidium iodide^−^ cells (bottom right quadrants). The percentage indicated in the quadrants of plots **D**, **E** and **F** refers to late apoptotic/necrotic cells (top right quadrants) and early apoptotic cells (bottom right quadrants) with activated caspase-8.

**Figure 4:**
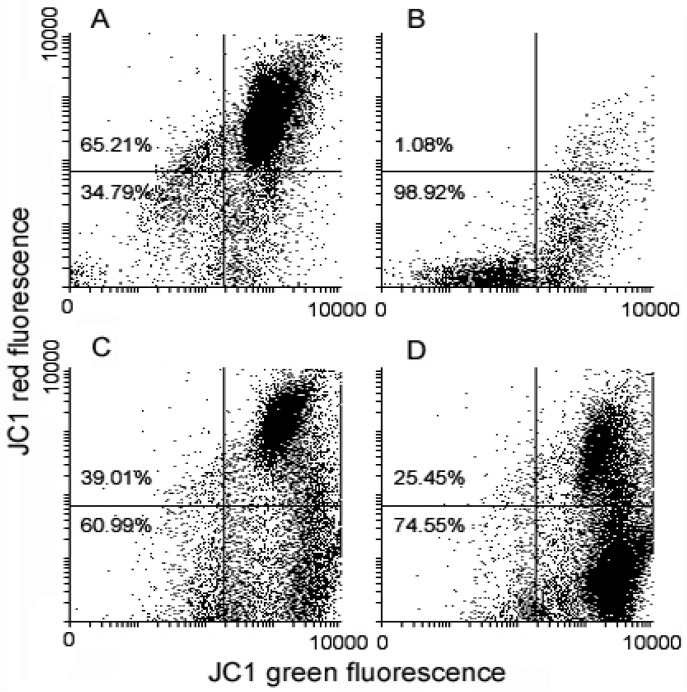
Flow cytometric analysis of untreated (**A**), valinomycin-treated (positive control) (**B**), **1/2** *cocktail*–treated (**C**) and **3**-treated (**D**) MDA-MB-231 cells stained with JC1 after 72 h of exposure for mitochondrial transmembrane potential (MMP) evaluation. The percentage in the bottom quadrants in each frame relates to low red-emitting cells that underwent MMP dissipation.

Literature reports indicate that drug-induced G_2_/M arrest of MDA-MB-231 cells is consistently associated with apoptosis promotion (e.g., [13]); on the other hand, an increase of the sub-G_0_/G_1_ cell fraction, consistent with the occurrence of apoptosis-triggered fragmentation of DNA, was observed at the left of the G_0_/G_1_ peak in both treated conditions. To assess if the cytotoxicity of the **1/2** *cocktail* and **3** were to be ascribed, at least in part, to the onset of programmed cell death, control and exposed cells were submitted to flow cytometric evaluation of apoptosis and mitochondrial metabolism markers. The panel in Figure 3 shows that, compared to controls, exposure to the drugs was associated with an increase of annexin V^+^/propidium iodide- (Figure 3A) and activated caspase-8+ (Figure 3B) apoptotic cells. In particular, **3** appeared to be more effective than the **1/2** *cocktail* in promoting phosphatydilserine externalization (**3** vs. **1/2** *cocktail* vs. control = 63.85% vs. 8.21% vs. 0.03%), whereas the extent of the enzyme activation between the two experimental conditions was more comparable (**3** vs. **1/2** *cocktail* vs. control = 24.66% vs. 20.83% vs. 1.34%). Variations of MMP after cell exposure to the drugs were detected using the JC1 probe. As shown in Figure 4, flow cytometry analysis suggests a loss of MMP in treated cells, in particular **3**-exposed cells to a higher extent, with the percentage of low red-emitting cells (bottom quadrants) being about 61% and 75% after 72 h of exposure to the **1/2** *cocktail* and **3**, respectively, vs. approximately 35% of control cells.

**Figure 5:**
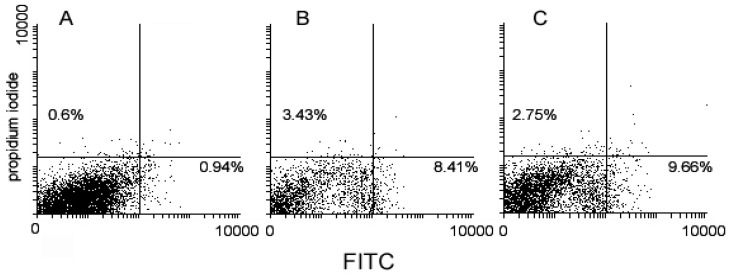
Flow cytometric analysis of untreated (**A**), **1/2** *cocktail*–treated (**B**) and **3**-treated (**C**) MDA-MB-231 cells stained with two-color reactive oxygen species (ROS) detection reagent after 72 h of exposure for MMP evaluation. The percentage indicated in the bottom right quadrants refers to total ROS overproducing cells, whereas that in the top left quadrants to superoxide anion–overproducing cells.

The ability of the drugs to affect the mitochondrial metabolism was also checked by assaying ROS production using a commercial kit, which differentiates between total ROS and superoxide ions. As shown in Figure 5, the dissipation of MMP was mirrored by an enhanced production of ROS (**3** vs. **1**/**2** *cocktail* vs. control = 9.66% vs. 8.41% vs. 0.94%) including a more moderate increase of the superoxide anion (**3** vs. **1**/**2** *cocktail* vs. control = 2.75% vs. 3.43% vs. 0.6%).

**Figure 6:**
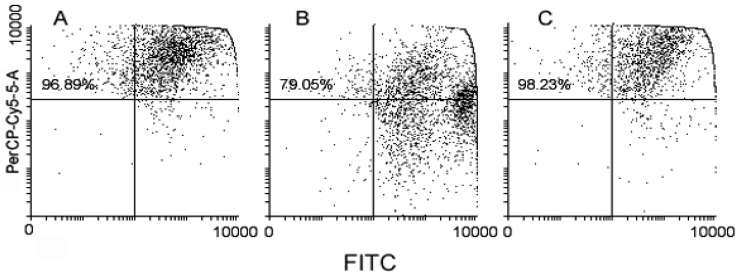
Flow cytometric analysis of untreated (**A**), **1/2** *cocktail*–treated (**B**) and **3**-treated (**C**) MDA-MB-231 cells stained with acridine orange after 72 h of exposure for evaluation of acidic vesicular organelle (AVO) accumulation. The percentage in the top quadrants relates to AVO-positive cells.

It has been reported that MDA-MB-231 cells have a constitutively high autophagy rate [14], thereby providing cells with energy and basic elements to counterbalance the metabolic stress associated with hypoxia and nutrient shortage and fast proliferation. It is also acknowledged that the inhibition of autophagy sensitizes MDA-MB-231 tumor cells to the lethal effect of chemical and physical agents (e.g., [15,16]). Therefore, in a last set of analyses, we checked whether **1/2** *cocktail* and **3** might induce a modification of the amount of autolysosomes, also known as AVOs, a hallmark of autophagy, through acridine orange staining. Interestingly, Figure 6 shows that **1/2** *cocktail*–treated cells underwent a consistent reduction of AVO accumulation, whereas their amount in **3**-treated cells was comparable to that of the control (**3** vs. **1/2** *cocktail* vs. control = 96.89% vs. 79.05% vs. 98.23%).

**Figure 7:**
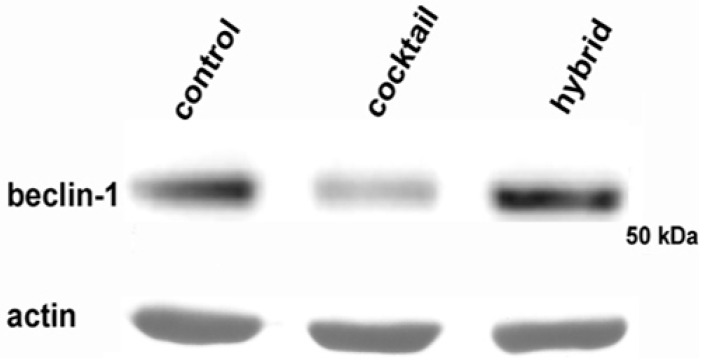
Western blot analysis of beclin-1. The image shows a prototypical example of a Western blot of total cell lysates after exposure of MDA-MB-231 cells to a **1/2** *cocktail* and **3** and analyzed with an antibody raised against beclin-1. Treatment with *cocktail* led to a reduction of the intensity of the beclin-1 band. Actin was immunostained as a loading control. The molecular weight marker is indicated on the right (Cruz Marker, Santa Cruz Biotechnology, Dallas, TX, USA).

The flow cytometric result was confirmed by a reduction in the amount of beclin-1, an essential mediator involved in autophagy machinery, in MDA-MB-231 cells exposed to the **1/2** *cocktail*, as visualized by Western blot (Figure 7). This revealed autophagy inhibition as a possible further aspect, alternative to apoptotic induction, the level of which was lower than in **3**-treated cells, involved in the cytotoxicity exerted by the drug *cocktail* on the TNBC cell line.

## 3. Discussion

In this paper, we have examined the cytotoxic effect of an HDACi (**1**) and a kinase inhibitor (**2**) on MDA-MB-231 cells, derived from a pleural effusion of a TNBC of the basal subtype and endowed with an “aggressive” phenotype in vivo [17]. We compared their biological activity in the form of both a 1:1 *cocktail* of the separate compounds and a chemically synthesized hybrid, at a concentration equal to their IC_50_ at 72 h, as already reported [3]. Several previous studies have confirmed the synergistic effects of drugs used in combination as potential anticancer agents. For example, Zhang et al. [18] have shown that a combination of Vorinostat (SAHA, **1**) and the antiangiogenic kinase inhibitor Sorafenib, acting on RAF kinase and the VEGFR-2/PDGFR-β pathway, exhibited in vivo synergism on death induction in different tumor cytotypes. Since their findings suggested the activation of the extrinsic apoptotic pathway, in order to assess the biological aspects of the cytotoxicity of the compounds under study, it prompted us to examine whether this could also be the mode of action of the **1/2** *cocktail* and/or **3**. Preliminarily, cell cycle analysis showed a perturbation of the rate of progression through the cycle, with aspects of redistribution of cells over different cycle phases for the two treatments, and a significant increase of the sub-G_0_/G_1_ cell population, indicative of DNA fragmentation which might occur in apoptotic cells, after exposure to both the *cocktail* and the hybrid. The annexin-V assay confirmed that apoptosis was promoted by exposure to the drugs, more prominently in the case of hybrid **3**, and the observed caspase-8 activation was suggestive of the occurrence of receptor-mediated death signaling [19]. Interestingly, both treatments were effective in inducing the dissipation of MMP, also in this case more prominently in the presence of **3**, and augmenting the levels of ROS, which are classically considered as crucial events in the intrinsic mitochondria-mediated pathway of apoptosis [20]. It is currently acknowledged that caspase-8 may stimulate the mitochondrial pathway of apoptosis via the proteolytic maturation of BH3 interacting-domain death agonist (BID) protein, thereby promoting the permeabilization of the outer mitochondrial membrane and the release of cytochrome c [21,22]. On the other hand, the co-existence of both apoptotic pathways in MDA-MB-231 after treatment with a plant metabolite has been reported [23]. Thus, whether exposure especially to **3** induces apoptosis in MDA-MB-231 cells by converging the death receptor-mediated extrinsic and the mitochondrial intrinsic pathways, although plausible, remains to be determined through further assays. Nevertheless, our cumulative results imply a higher efficacy of **3** in triggering programmed cell death on TNBC cells.

Autophagy is a cellular homeostatic function in which cells autodigest cytoplasmic substrates for removal or turnover after sequestration in multi-membrane-bound structures, the autophagic vacuole, and subsequent fusion with lysosomes generating AVOs or autolysosomes [24]. It plays a complex and highly controversial role in breast cancer: on one hand, autophagy can result in cell death, thereby acting as a tumor-suppressor mechanism, but on the other hand, it can exert a cell-protective role via intracellular recycling, providing energy and basic elements which allow tumor cell survival in stress conditions of oxygen and nutrient shortage and rapid proliferative rate [25]. The latter pro-survival role appears to be the effect of the constitutively-elevated autophagy rate of MDA-MB-231 cells [14]. In fact, various publications have demonstrated that inhibition of autophagy in this cell line leads to sensitization to the lethal effect of chemicals and apoptosis activation [14,26,27]. In this paper we have checked whether the drugs under study might modulate the autophagic rate by two different approaches, i.e., the flow cytometric evaluation of AVO accumulation in conjunction with the immunorevelation of the protein marker beclin-1 [28], which is a key component of autophagy machinery belonging to the signal-initiating class III phosphatidylinositol-3 kinase complex [29]. Interestingly, down-regulation of autophagy appeared to be most prominently triggered by the **1/2** *cocktail*, thus suggesting that the two forms of the examined compounds could induce cell death by different preferential pathways, i.e., autophagy inhibition (**1/2** *cocktail*) or apoptosis promotion (**3**).

It is noteworthy that a preliminary evaluation of the individual IC_50_ of either **1** or **2** after 72 h of exposure revealed a different potency of the two compounds on MDA-MB-231 cells (approximately 1 μM for **1** [30] and 100 μM for **2**), thereby indicating that equipotent co-treatment could have been performed with a 1:100 **1/2** *cocktail*. Surprisingly, this formulation was not able to induce more than about 20% cell loss at the highest concentrations tested (Luparello, unpublished data) and therefore an equimolar mixture of the drugs (10 μM for both), i.e., the same used in our previous publication [3], was used in the present study. Thus, it can be hypothesized that excess **1** might be responsible for the more marked G_2_/M arrest and involvement of apoptosis induced by treatment with 1:1 **1/2** *cocktail*, in light of previous studies on the effects of SAHA on MDA-MB-231 cell cycle distribution [31] and viability reduction due to activation of programmed cell death [32,33]. In particular, consistent with our findings from these studies, both apoptotic pathways appeared to be triggered, since the studies [32] demonstrated the activation of the intrinsic apoptotic pathway and the occurrence of caspase-3 cleavage, whereas others [33] reported the upregulation of genes associated with the extrinsic apoptotic pathway, such as TNF-related apoptosis-inducing ligand (TRAIL) and caspase-8 among the others.

In conclusion, our results further substantiate the impressive potential of the hybrid vs. combination approach in finely tuning the biological activities of target cells and also strongly encourage the further optimization of **3**-like molecules to develop a promising additional prevention and/or treatment agent effective against “aggressive” breast carcinomas, such as TNBC.

## 4. Materials and Methods

### 4.1. Cell Culture and Drug Treatments

MDA-MB-231 breast cancer cells, were available in laboratory stocks and already used for study in our lab (e.g., [14]), were maintained in a RPMI 1640 medium (Sigma, St. Louis, MO, USA) supplemented with 10% (*v*/*v*) fetal bovine serum (FBS) and 1% antibiotics (*v/v*) (100 U/mL penicillin, 100 μg/mL streptomycin and 2.5 mg/L amphotericin B (Invitrogen, Carlsbad, CA, USA)) in a humidified atmosphere at 37 °C in 5% CO_2_. Cells were detached from flasks with 0.05% trypsin-EDTA, counted, and plated at the required density for treatment once 60%–80% confluency was attained.

The HDACi **1** was purchased from Selleck Chemicals, compounds **2** and **3** were synthesized as reported [34].

In light of the values of IC_50_ at 72 h already reported for 1:1 **1**/**2** *cocktail* and for **3** [3], the biological assays were performed in the presence of either 10 μM of the **1**/**2** *cocktail* or 29 μM of **3**. Control cells were exposed to dimethyl sulphoxide, (Santa Cruz Biotechnology, Dallas, TX, USA) at the same concentrations.

### 4.2. Flow Cytometry

Flow cytometric assays were performed according to Librizzi et al. [14].

MMP was verified by use of a fluorescent dye JC1 (Molecular Probes, Eugene, OR, USA), which is selectively taken up into mitochondria, leading to a fluorescence emission shift from green (~529 nm) to red (~590 nm) for intact MMP, although in the case of mitochondrial depolarization a decrease in the red/green fluorescence intensity ratio is observed. The ionophore valinomycin (Sigma), which induces mitochondrial gradient dissipation, was co-incubated at 1 μM concentration with JC1 for positive control. Data were represented as dot plots using Flowing Software v.2.5.1. (Mr. Perttu Terho, Turku Centre for Biotechnology, Turku, Finland), which discriminate, in the bottom quadrants, the amount of cells undergoing a loss of MMP.

Quantification of AVOs was obtained via acridine orange staining (Sigma, final concentration of 100 μg/mL) for 20 min (in the dark) prior to analysis. Data are displayed as dot-plots using the Flowing software v.2.5.1., which is able to discriminate cells with increased AVO accumulation in the top quadrants.

Cell cycle distribution was checked using propidium iodide stain following pre-incubation with Triton X-100 and RNase A (Sigma), and analyzed with Weasel v.3.0.1. software (Murray Jeffs, Walter & Eliza Hall Institute of Medical Research, Parkville, Australia).

Activation of caspase-8 was assessed with a Vybrant FAM caspase-8 assay kit (Molecular Probes) following the manufacturer’s instructions. Data are represented as dot-plots with Flowing software v.2.5.1., which discriminates early and late apoptotic cells with increased enzyme activation in the bottom and top right quadrants, respectively.

All the preparations assayed contained both attached and floating cells, and all the analyses were carried out in a FACSCanto apparatus (BD Biosciences, Franklin Lakes, NJ, USA).

### 4.3. Western Blot

Electrophoretic analyses and immunoblots were carried out according to Librizzi et al. [35].

## Abbreviations

HDACi	histone deacetylase inhibitor
SAHA	suberoylanilide hydroxamic acid
VEGFR1/2i	vascular endothelial growth factor-1 and -2 receptor inhibitor
TNBC	triple-negative breast cancer
EGFR	epidermal growth factor receptor
VEGFR	vascular endothelial growth factor receptor
PI3K	phosphatidylInositol 3-kinase
STAT3	signal transducer and activator of transcription 3
IC_50_	half maximal inhibitory concentration
MMP	mitochondrial transmembrane potential
ROS	reactive oxygen species
AVO	acidic vesicular organelle
PDGFR	platelet-derived growth factor receptors
BID	BH3 interacting-domain death agonist
TRAIL	TNF-related apoptosis-inducing ligand
FBS	fetal bovine serum
EDTA	ethylenediaminetetraacetic acid
SDS-PAGE	sodium dodecyl sulphate-polyacrylamide gel electrophoresis
